# In honor of Federico Capasso, a visionary in nanophotonics, on the occasion of his 75th birthday

**DOI:** 10.1515/nanoph-2025-0449

**Published:** 2025-11-07

**Authors:** Alexandra Boltasseva, Nader Engheta, Giuseppe Strangi, Dennis Couwenberg

**Affiliations:** Elmore Family School of Electrical and Computer Engineering, Birck Nanotechnology Center, and Purdue Quantum Science and Engineering Institute, Purdue University, West Lafayette, IN 47907, USA; Quantum Science Center, Oak Ridge National Laboratory, Oak Ridge, TN 37830, USA; 6572 University of Pennsylvania, Philadelphia, PA, USA; Department of Physics, NLHT-Lab, University of Calabria, Arcavacata, Italy; CNR-NANOTEC, Institute of Nanotechnology, 87036, Rende, Italy; Department of Physics, Case Western Reserve University, Cleveland, OH, 44106, USA; Marie Heinekenplein 404, 1072 ML, Amsterdam, The Netherlands

The field of nanophotonics has been blessed by the groundbreaking and transformative contributions of our dear friend, Professor Federico Capasso, who is a brilliant scientist, an ingenious innovator, and an outstanding inspiration to all of us. Over several decades of scientistic activities, he has made trailblazing and monumental contributions and breakthroughs in many branches of science and technology of light–matter interaction, with very broad and at the same time very deep and fundamental impacts on various fields. His pioneering work includes an amazing list of topics such as quantum cascade laser, bandgap engineering, metasurfaces, generalization of Snell’s law, Casimir force measurement, organic transistors, bound state in the continuum, polarimetry, bio-inspired imaging, and many more. In each of these topics, he has been at the forefront and has done pioneering work. In addition to his wonderful scientific work, Professor Capasso has also been very active in technology transfer by bringing some of his scientific findings to the market by co-founding two successful companies, EOS Photonics in 2010 for his work on QCL, and Metalenz in 2016 for his work on metasurfaces. He has received numerous prestigious awards and has been elected to several national academies, each of which represents his pioneering and groundbreaking work. He is a great role model for all of us.

To honor Professor Federico Capasso as a pioneer in the field of nanophotonics, and also on the occasion of his 75th birthday, this special issue is dedicated to him and his outstanding achievements to date. In addition to 47 invited papers, we have collected anecdotes and photographs from friends and colleagues celebrating his fruitful collaborations, expert mentorship, and research leadership.

**Alexandra Boltasseva**

**Nader Engheta**

**Giuseppe Strangi**

Guest Editors

**Dennis Couwenberg**

Managing Editor

## Anecdotes:

**Dennis Couwenberg**

*Publishing Editor, Nanophotonics*

It was my first job and I remember my first meeting with Federico. I was 25 and working for Elsevier on the journal *Optics Communications* that was originally owned and published by the North Holland Publishing Company before Elsevier took it over in 1970. We talked about publishing and I was surprised about his open and welcoming attitude, his enthusiasm for science and his ideas about publishing. He mentioned that North Holland Publishing was a highly reputable Dutch publishing house that was known for their reputable scientific journals with high scientific standards. It was one of the conversations that inspired a spark of curiosity in me and opened my mind to aim for higher scientific standards in publishing ever since.

Now 15 years ago Federico founded the journal Nanophotonics. Looking back, one can see how his values are an integral part of the journal’s foundation and its success. His drive for high standards, a heartfelt support for young scientists and a passion for out of the box science turned life changing technology. It comes back in the high journal standards we set out 15 years ago for the papers published, the charity issues we pioneered, and the journal’s many prizes and certificates that are awarded to young scientists every year.

Thank you for inspiring me to look at science and do scientific publishing differently.

My gratitude goes beyond words for all these years of collaboration, inspiration and connection.

Dennis

**Figure:**
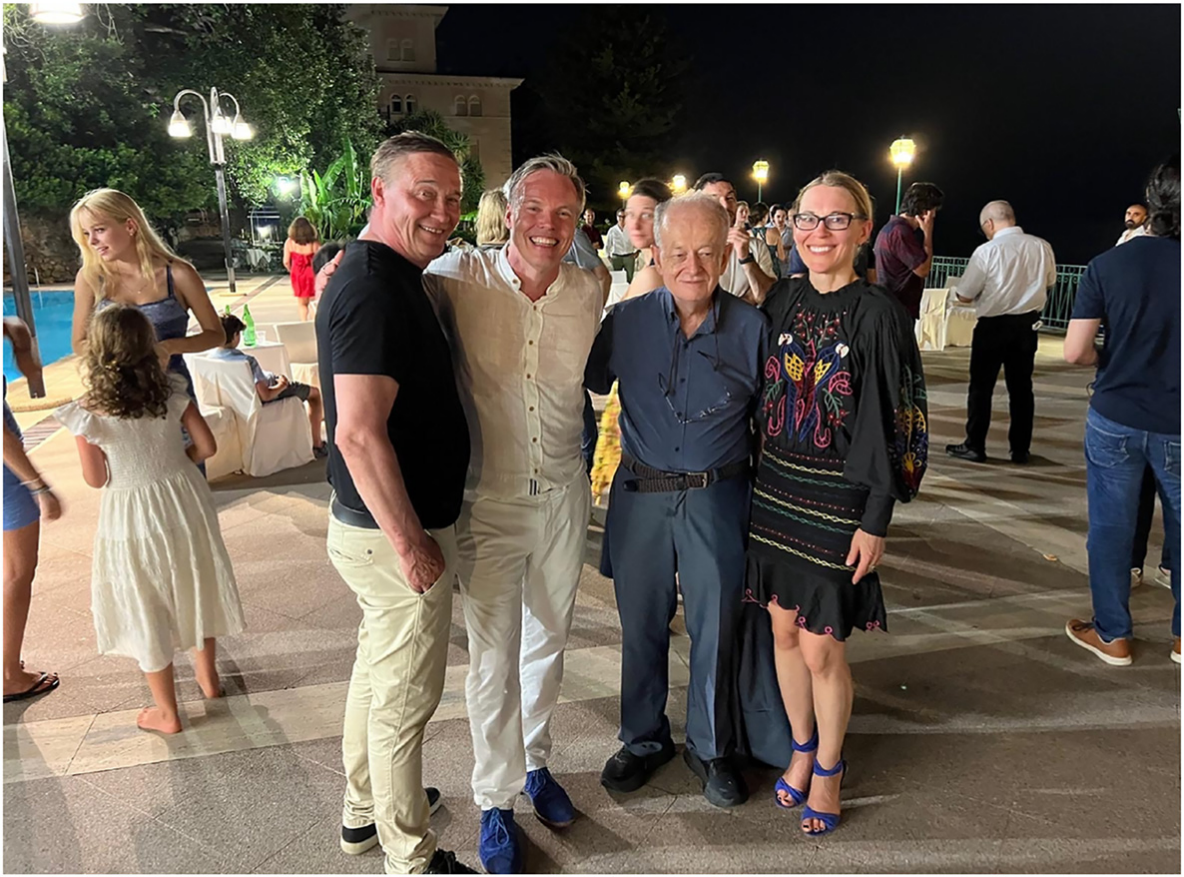


**Figure:**
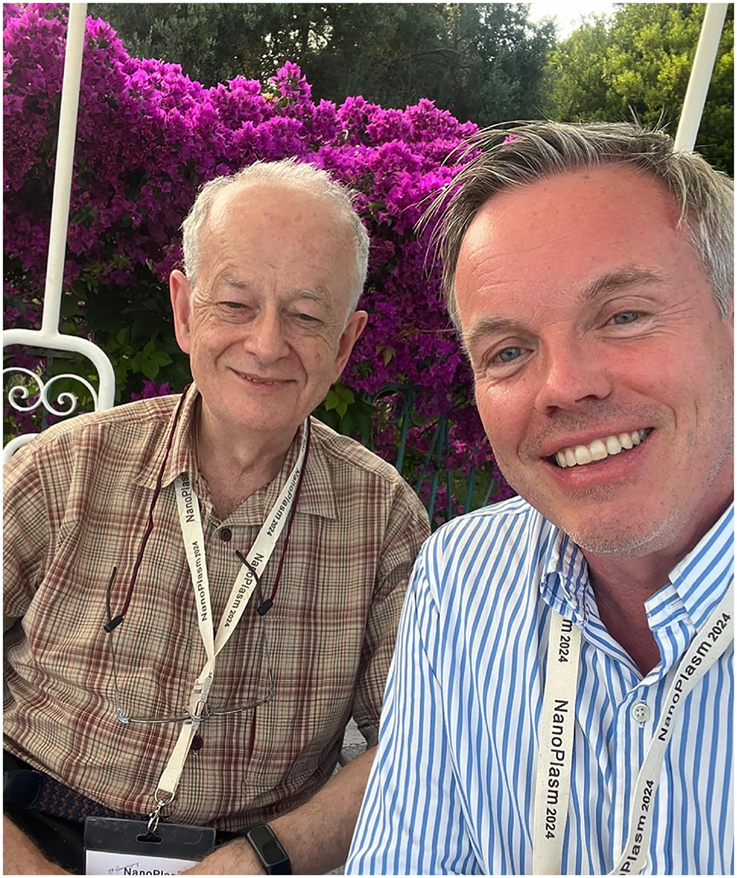


**Alexandra Boltasseva and Vladimir Shalaev**

*Guest Editor | Purdue University, USA*

**Figure:**
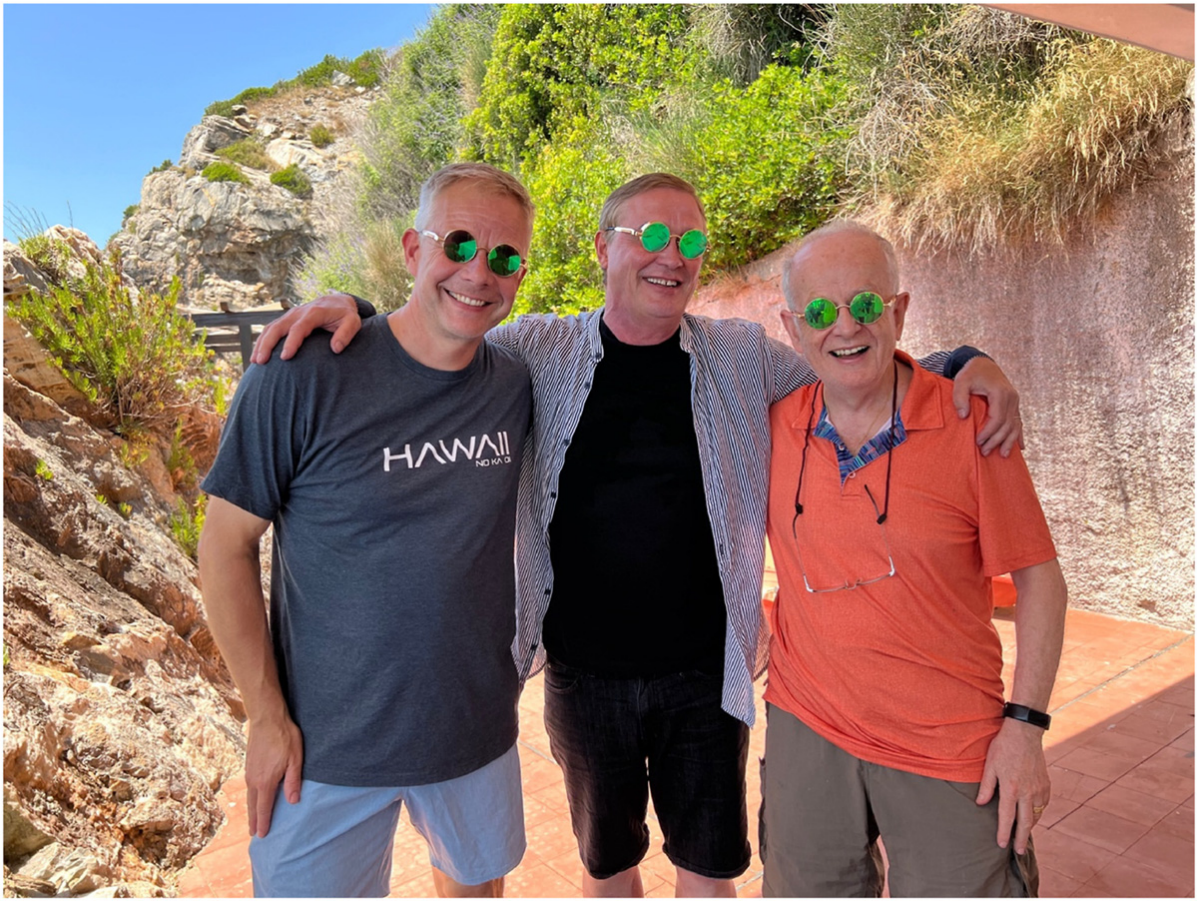


**Figure:**
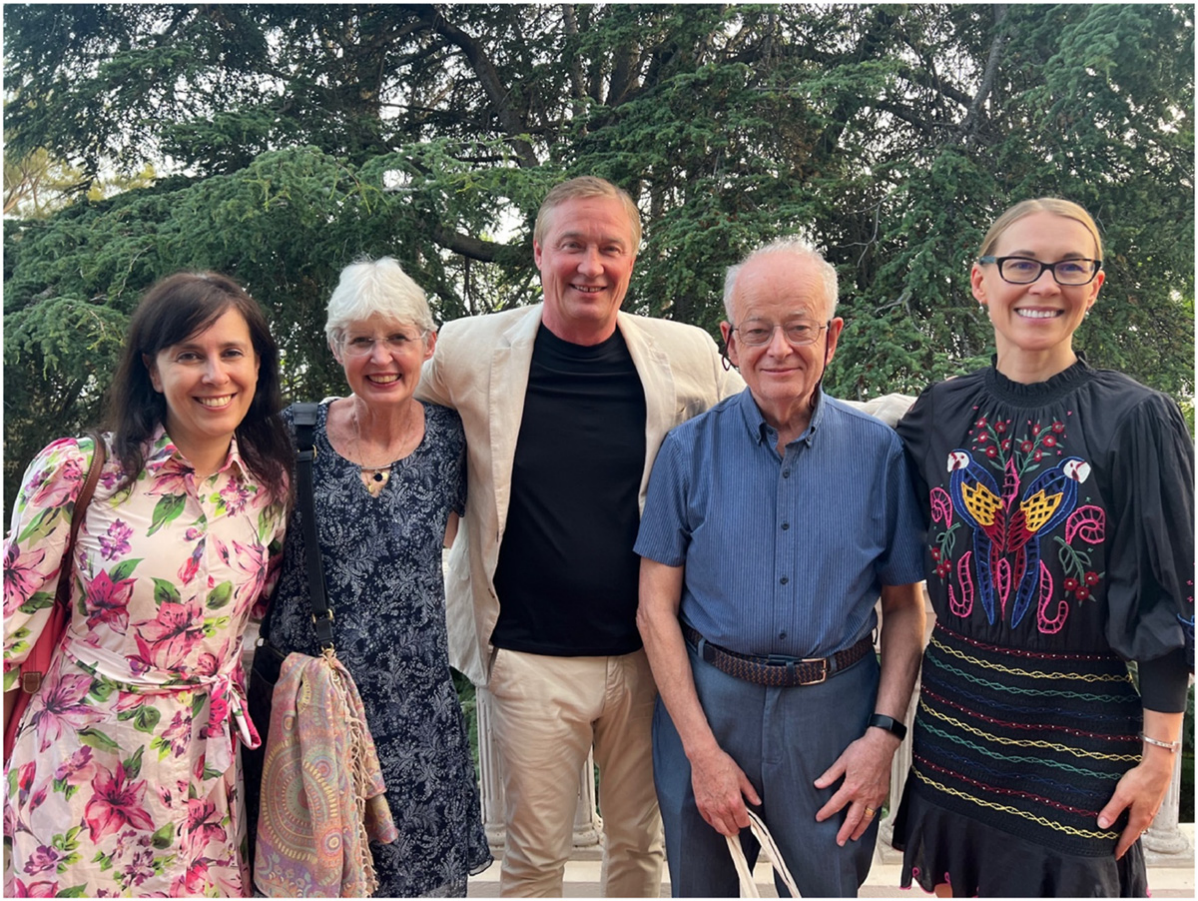


**Nader Engheta**

*Guest Editor | University of Pennsylvania, USA*

**Figure:**
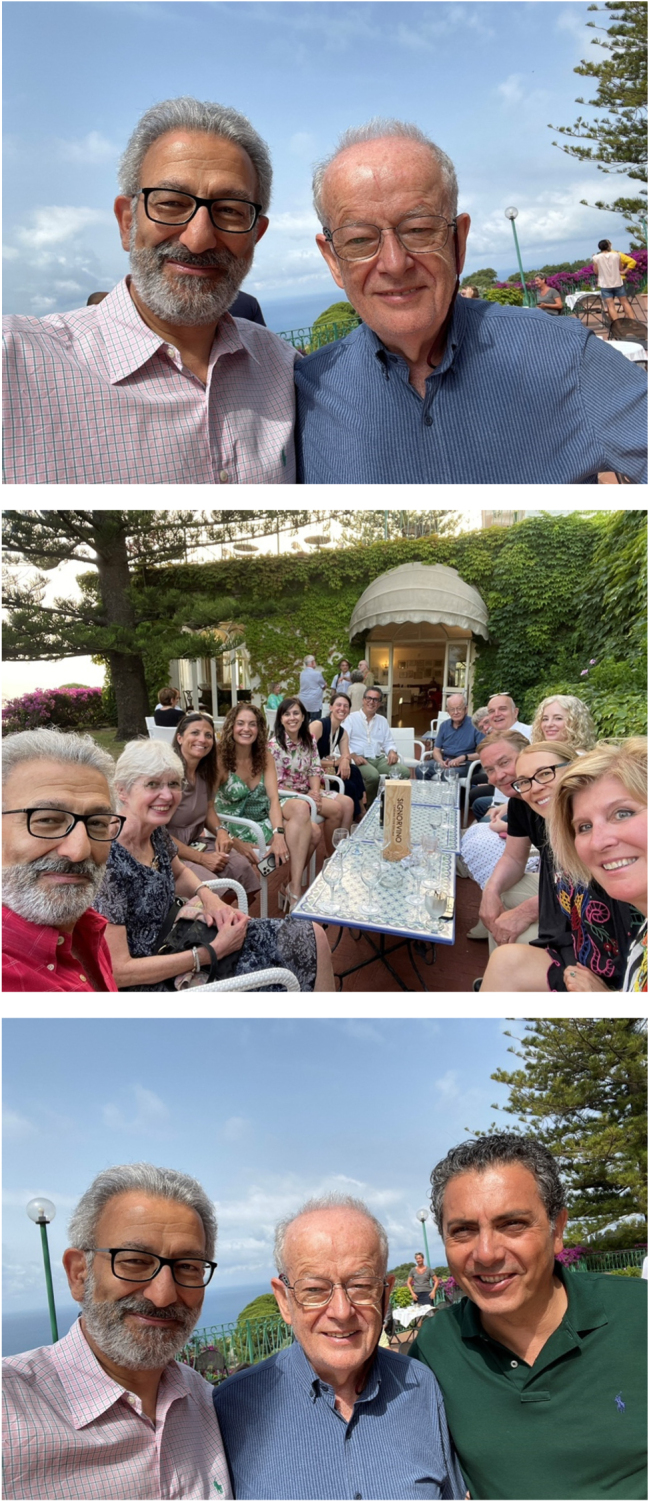


**Giuseppe Strangi**

*Guest Editor | Case Western Reserve University, USA*

I first heard about Federico long before I met him – back in Rome, some 30 years ago, when his name was already orbiting through the physics world like a comet. By the time I finally met him, he had already moved to Harvard. We began with conversations, then collaborations, often on physics problems that were as bizarre as they were fascinating. Federico has always had a special radar for the bizarre – meeting each idea with what he calls his “paranoid” attitude. After all, his motto has always been: “Only the paranoids survive.” In 2018, Paola, Federico, my wife Antonella, and I took a short trip to Matera, that year’s European Capital of Culture. Traveling with Federico is like carrying an entire Encyclopedia Britannica in your pocket. Museums, restaurants, architecture, even the physics of a 4000-year-old city – nothing escapes his curiosity. We keep collaborating, and my students still tremble at the thought of presenting results to him – because he always finds that question or insight that you never saw, but that was staring you in the face the whole time. Federico is also disarmingly funny, easygoing, and – believe it or not – an excellent dancer. If you need proof, just come to Nanoplasm 2026 in Italy, where he will surely lead us all in his legendary performance of “the chicken dance”.

**Figure:**
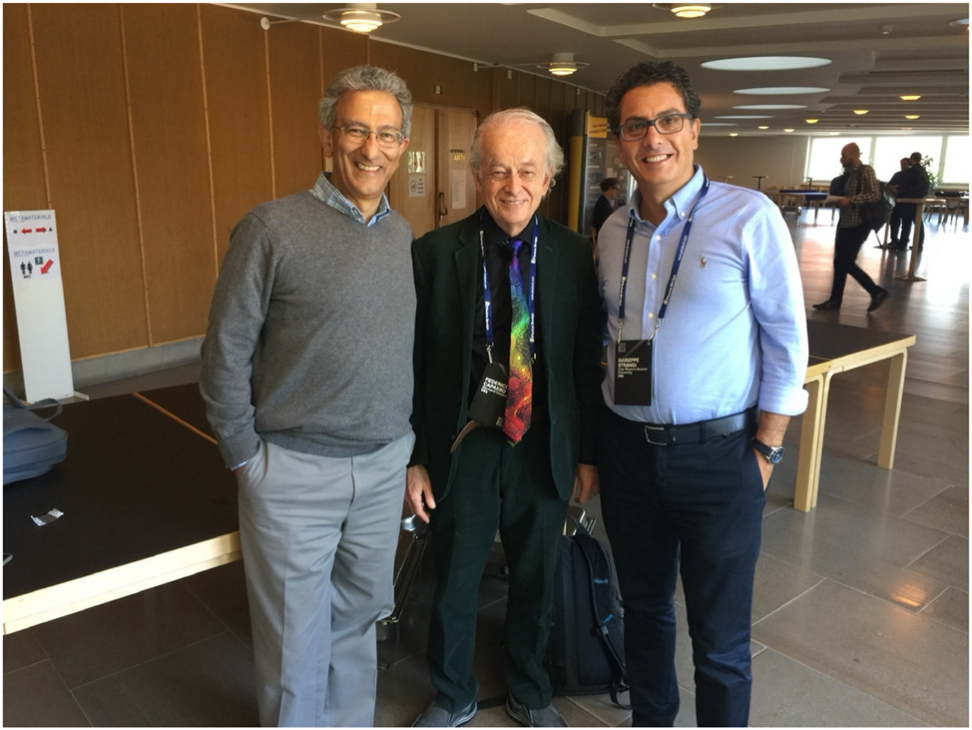


**Hatice Altug**

*EPFL, Switzerland*

I first learned about Federico’s profound scientific impact as an undergraduate student in Turkey while studying quantum physics for the first time. Reading his paper on quantum cascade laser (QCL) was very exciting, as it provided a perfect textbook example of how quantum engineering and materials science can come together to revolutionize technology. The invention of the QCL represents a landmark achievement, delivering a highly tunable, powerful, and coherent light source operating in the mid-infrared spectral range. Many molecules exhibit their characteristic absorption fingerprints in this spectral range, therefore mid-IR spectroscopy enables chemically specific, non-destructive sample analysis, making it invaluable for applications in biochemical research, medical diagnostics, pharmaceuticals, environmental monitoring, remote sensing, and security. During my early years as an assistant professor at Boston University, I was fascinated by Federico’s pioneering work combining nanoplasmonic antennas with QCLs for advanced beam shaping. Another milestone contribution he made in nanophotonics field is the concept of the metasurface, which led to the development of flat optics and numerous innovative wavefront-shaping strategies using plasmonic and dielectric nanostructures. I vividly recall a conference talk where he emphasized the importance of “interfaces” and the potentials of going beyond bulk materials with “designer materials”, which he perfectly realized with QCLs and metasurfaces to control electrons and photons in new ways. These groundbreaking inventions from Federico’s lab have significantly influenced my research interests, particularly for developing mid-IR metasurfaces for surface-enhanced infrared absorption (SEIRA) spectroscopy and applying them to biomedical applications. For instance, we demonstrated the integration of engineered plasmonic metasurfaces with microfluidic systems and AI techniques to perform real-time mid-IR spectroscopy measurements in aqueous solutions, enabling the analysis of heterogeneous samples containing diverse biomolecules using their molecular-specific IR spectra [1]. In another study, we utilized SEIRA biosensors to distinguish between different conformational states of the same protein and detect misfolded toxic aggregates, which is highly relevant for neurodegenerative diseases [2]. Additionally, we introduced dielectric metasurfaces for imaging based fingerprint detection, leveraging on-chip mid-IR sources like QCLs [3], [4]. We have an upcoming collaborative review article with Federico and other leading pioneers, on the transition of optical metasurfaces from scientific exploration to technological innovation – an endeavor that continues to inspire me. I am thrilled by the rapid advancements in nanophotonics and mid-IR research and enthusiastic about their immense potential to transform into impactful real-world technologies.

**Andrea Alù**

*City University of New York, USA*

As an Italian scientist working in the US, and having grown up and studied in Roma, Prof. Capasso has been a true role model and inspiration since the start of my career. While I have not worked directly with him, I have collaborated with Prof. Capasso and his group on a few funded projects, and I have always been inspired by his depth, rigor and vision in science. Prof. Capasso continues impacting our community with his vision and remarkable contributions, year after year. I am very glad to contribute to this Honorary Issue, and I look forward to many more interactions with Prof. Capasso.

**Antonio Ambrosio**

*Istituto Italiano di Tecnologia (IIT), Italy*

*Teaching through self*

It was late Spring 2013. I met with Prof. Capasso in his office to discuss the status of a project that would also potentially lead to the first paper together. It was me, a group’s postdoc and Federico. Three chairs around, in a circle.

We (me and the postdoc) entered the office with the wrong assumption that Federico would not necessarily care too much about this study since It was really far (seriously far) from any other project going on in the group at the time. It was in fact something that casually happened to me, together with the other guy.

The discussion was intense, with Federico going deeply in any crucial passage, asking tons of questions. Soon, I realized not only that what we were presenting was not clear enough but also that we could not provide any decent answer, deep enough to cover the good points Federico was raising.

At some point a visibly frustrated Federico looked at me and said: “Antonio. Am I the only one here not to get such a description?”. I felt ashamed. Not because of any tension in the room. There wasn’t actually any; it was quite informal as usual. I felt ashamed simply because by doubting himself, in fact, Federico made me realize that I should have asked myself those questions before and I should have reached a deeper understanding of what I was proposing if I wanted others to understand after me.

**Figure:**
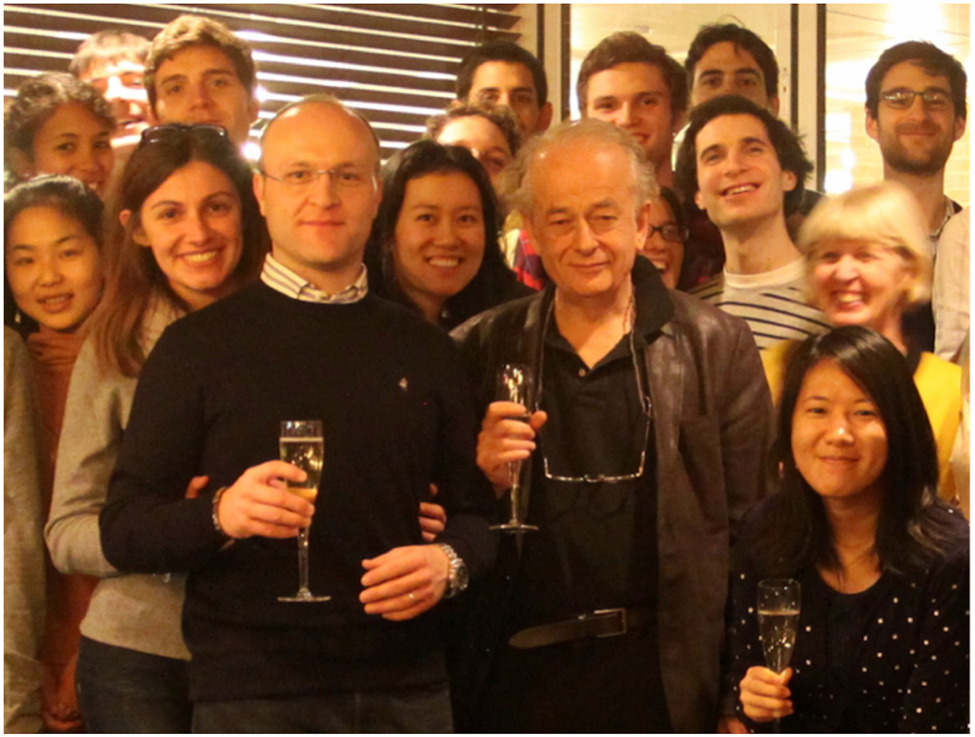


As I had the chance to experience tens of other times in the following seven years at Harvard, an infinite curiosity and passion for Science together with attention to details drove Federico’s research. I feel extremely lucky I had the chance to learn from him.

**Mikhail Belkin**

*Technical University of Munich (TUM), Germany*

**Figure:**
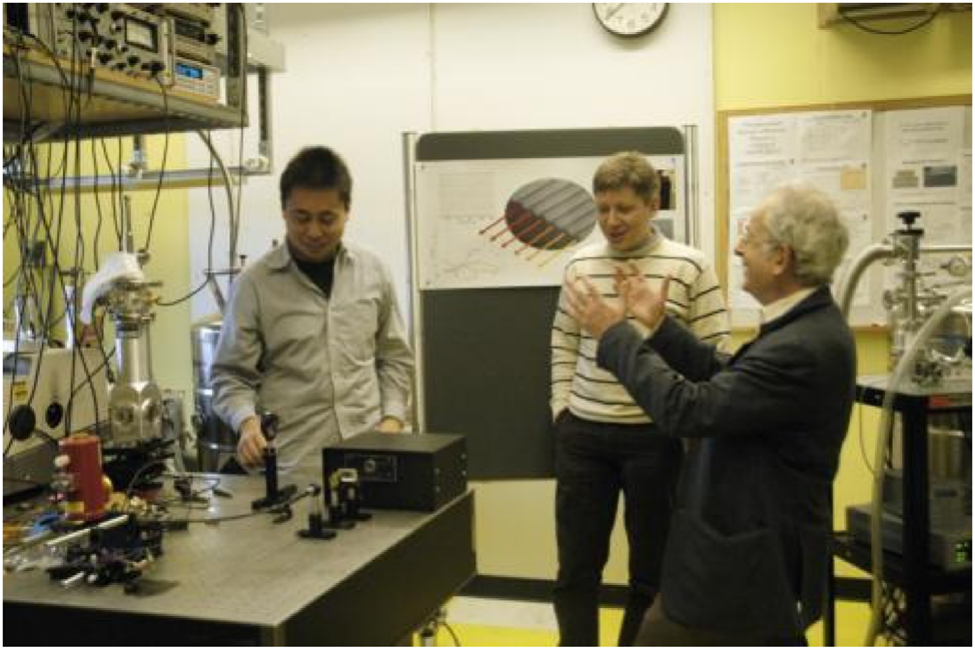


**Michele Celebrano**

*Polytechnic University of Milan, Italy*

**Figure:**
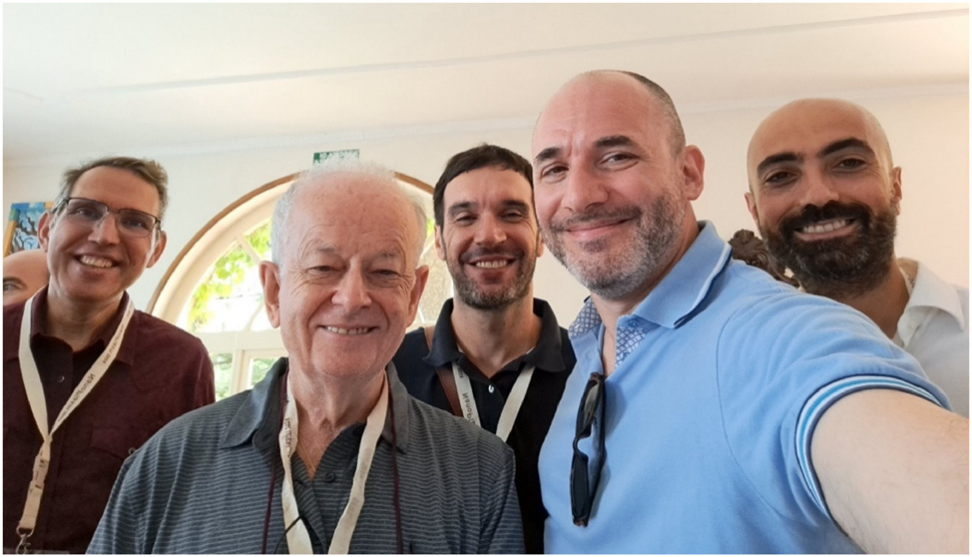


**Figure:**
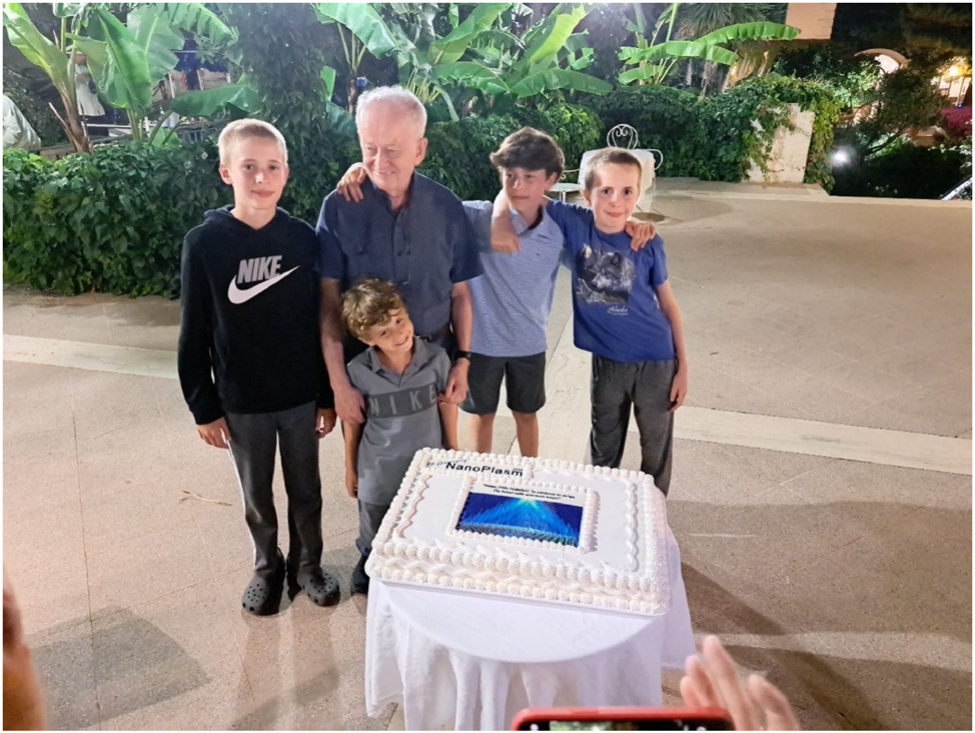


**Rob Devlin**

*Co-Founder and CEO, Metalenz*

**Figure:**
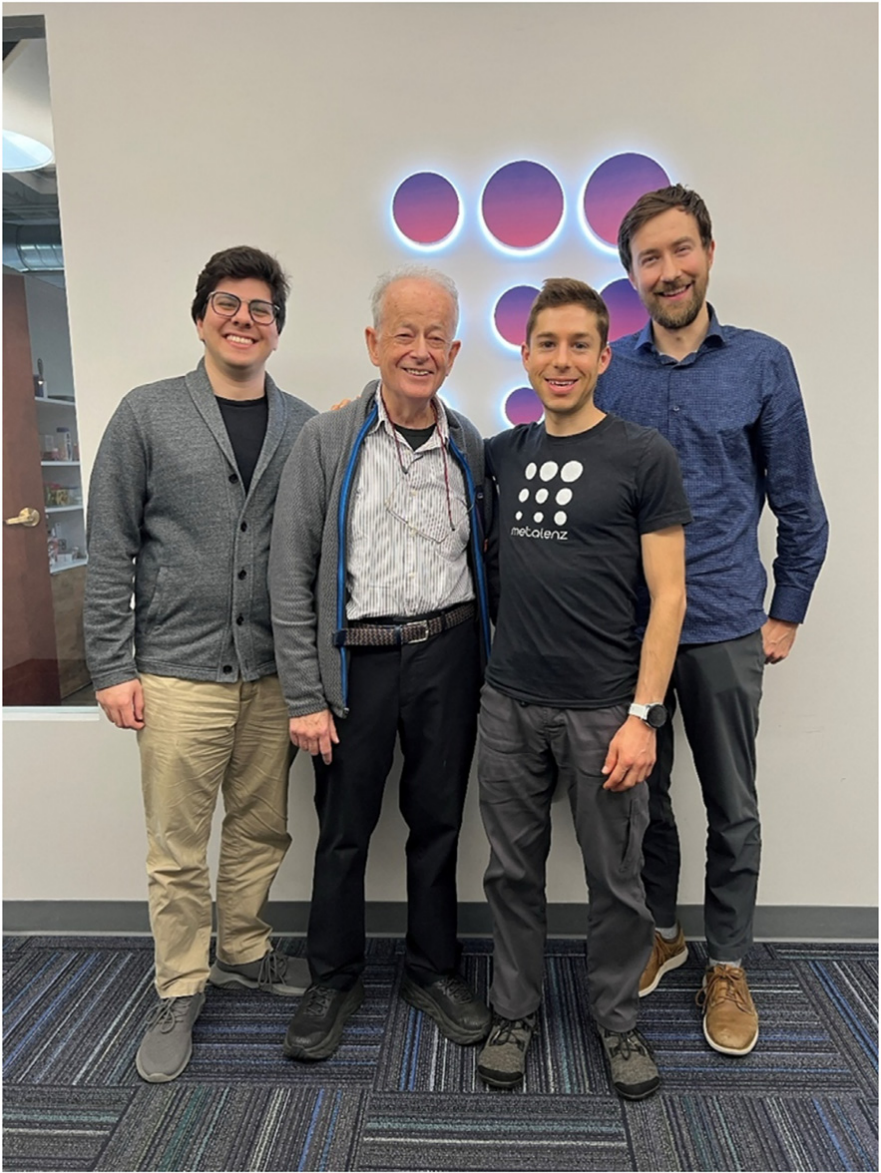


**Ahmed Dorrah**

*Eindhoven University of Technology, Netherlands*

I vividly remember my first handshake with Prof. Capasso in front of Pierce Hall on the Snowy afternoon of March 10th, 2019. My intimidation from joining such a pioneering research group as a postdoc quickly faded away through Prof. Capasso’s warm attitude, empowerment and trust. For the following five years, I had the privilege and pleasure to work closely with him and to flourish as a scientist and mentor. He instilled in us scientific curiosity, integrity and team spirit. He also broke many mental barriers, allowing our ideas and innovation to be set free. Sky indeed is the limit for all young researchers working in Capasso’s group where bright ideas supersede hierarchies. Besides research excellence, Prof. Capasso’s perseverance is a defining trait that not only shaped his extraordinary career but has also served as an inspiration to generations of scientists. I have many memorable moments (and voice notes) with Prof. Capasso, but I particularly cherish our group’s celebration of his 20th anniversary at Harvard University. The spark in Federico’s eyes as he shared glimpses from his remarkable career at Bell labs, recalling his legacy on QCLs and his later breakthroughs in flat optics was truly inspiring for all of us. I wish Prof. Capasso wonderful years ahead, filled with good health, happiness, and many more groundbreaking achievements!

**Figure:**
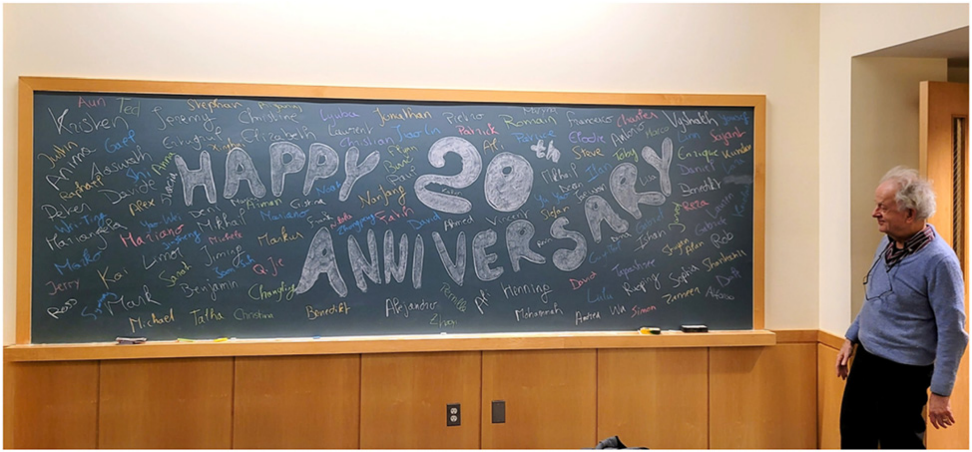


**Jérôme Faist**

*ETH Zürich, Switzerland*

The most important thing I learned from Federico was the value of expressing oneself correctly in publications. I remember being dead tired late at night – while sitting with Federico who was trying to find the “right word” to complete a sentence in a paper. There I realised that good data had to be matched with good wording.

**Figure:**
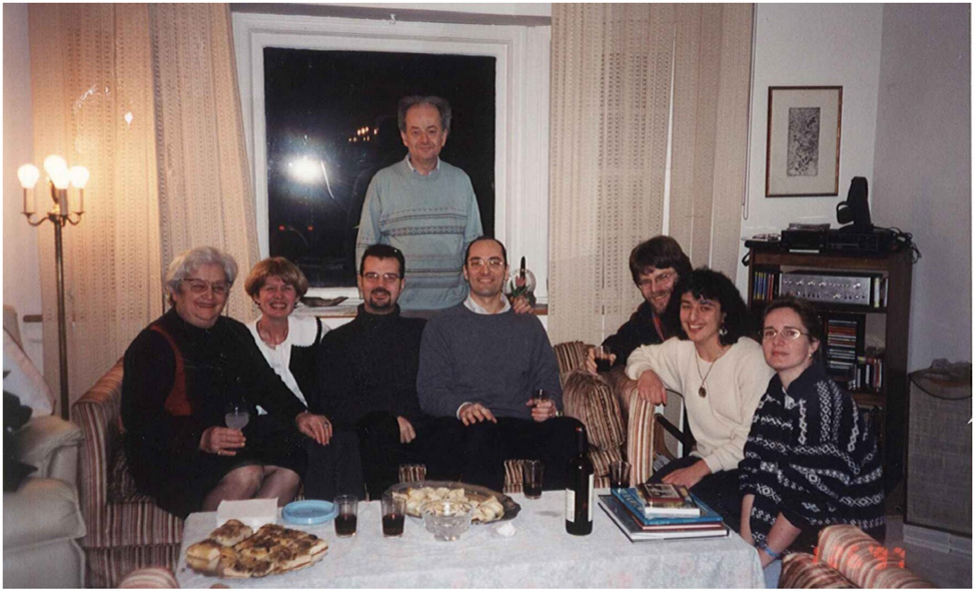


**Patrice Genevet**

*Colorado School of Mines, USA*

**Figure:**
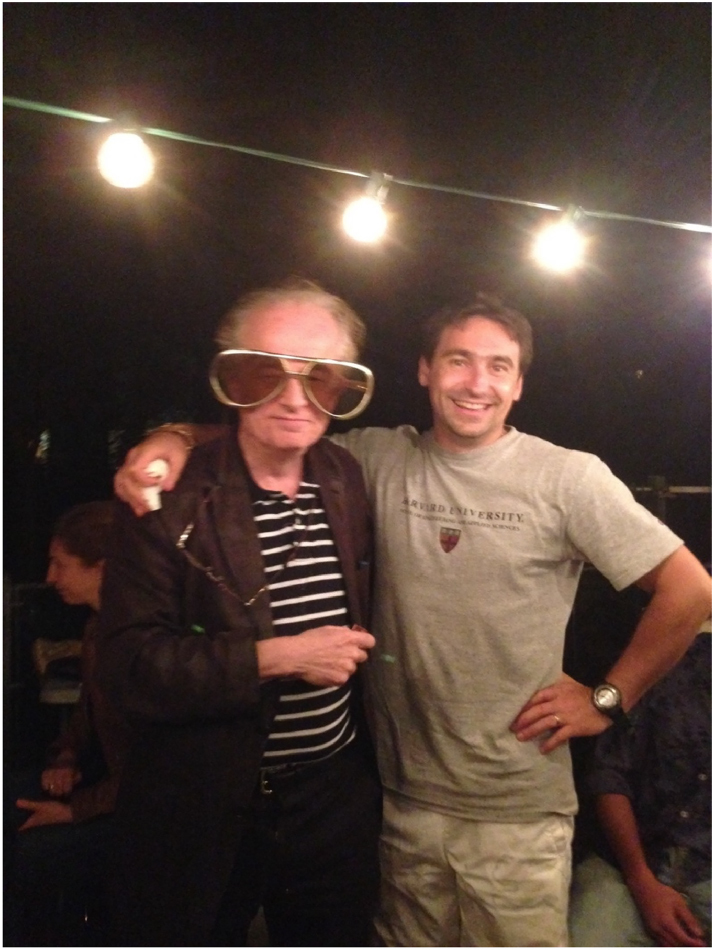


**Figure:**
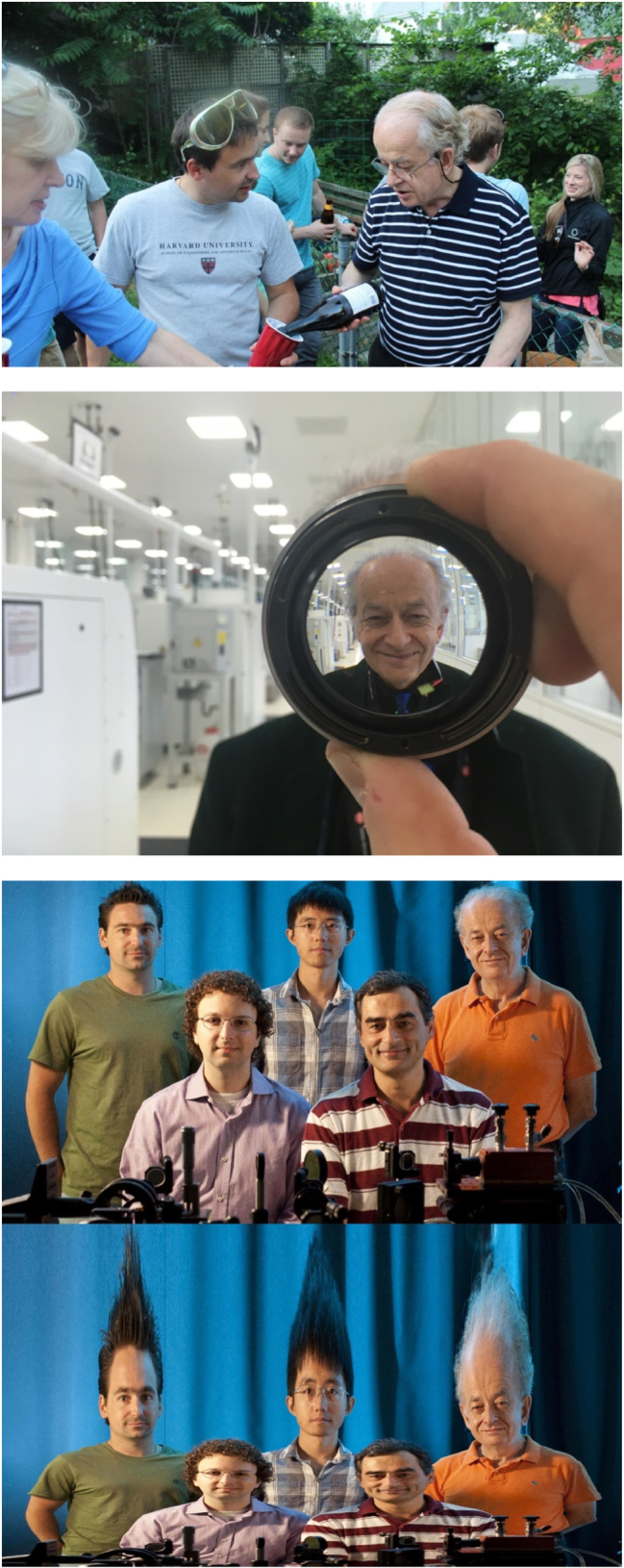


**Evelyn Hu**

*Harvard University, USA*

*Two Roads Diverged…and then Reconnected.*

Researchers in our field have a special good fortune of being able to share journeys of friendship and discoveries over many years, at the least sharing discoveries through publications, conferences, and collaborations. A particularly special gift is to be able to meet as young researchers, sharing aspirations and uncertainties about work and life, part ways geographically, though holding fast to common research fields, and then come together to a common setting again after many years. I was given that rare gift in my friendship with Federico and Paola, whom I met for the first time in 1976 at Bell Laboratories, Holmdel.

**Figure:**
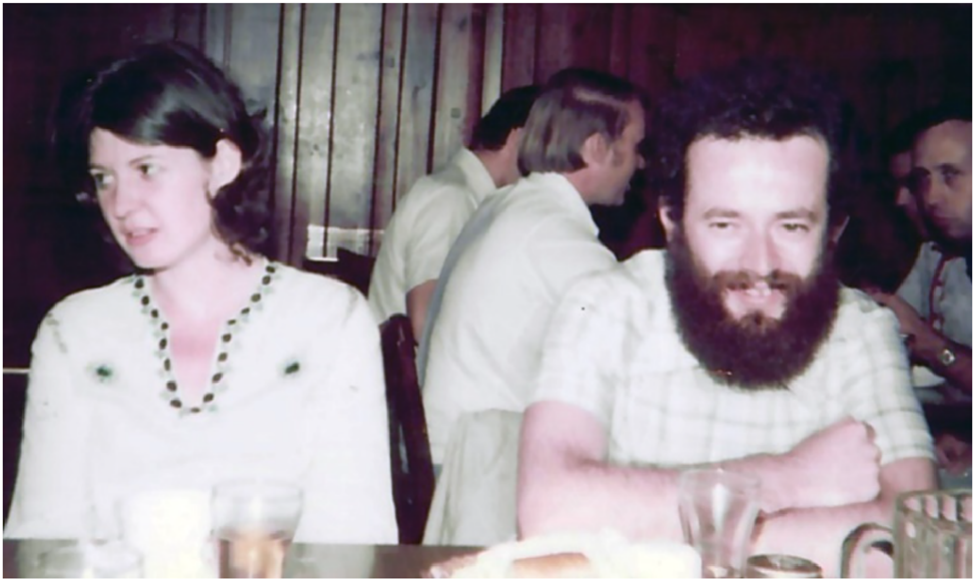


We worked in the same lab, under the directorship of P.K. Tien. More than that, we were neighbors, living in the area of New Brunswick, New Jersey, and so had time to share pizzas and picnics and wide-ranging political discussions, together with much laughter. Federico then and now balanced humor and love of life with a deep and strongly driven insistence on excellence, on foundational understanding, and on seeking the important problems. Thus, it was no surprise to me to learn of Federico’s research successes and the profound contributions of his work on quantum cascade lasers, although when these discoveries came to light, I was on the other side of the country, at USCB, and the day-to-day in-person interactions were no longer possible.

**Figure:**
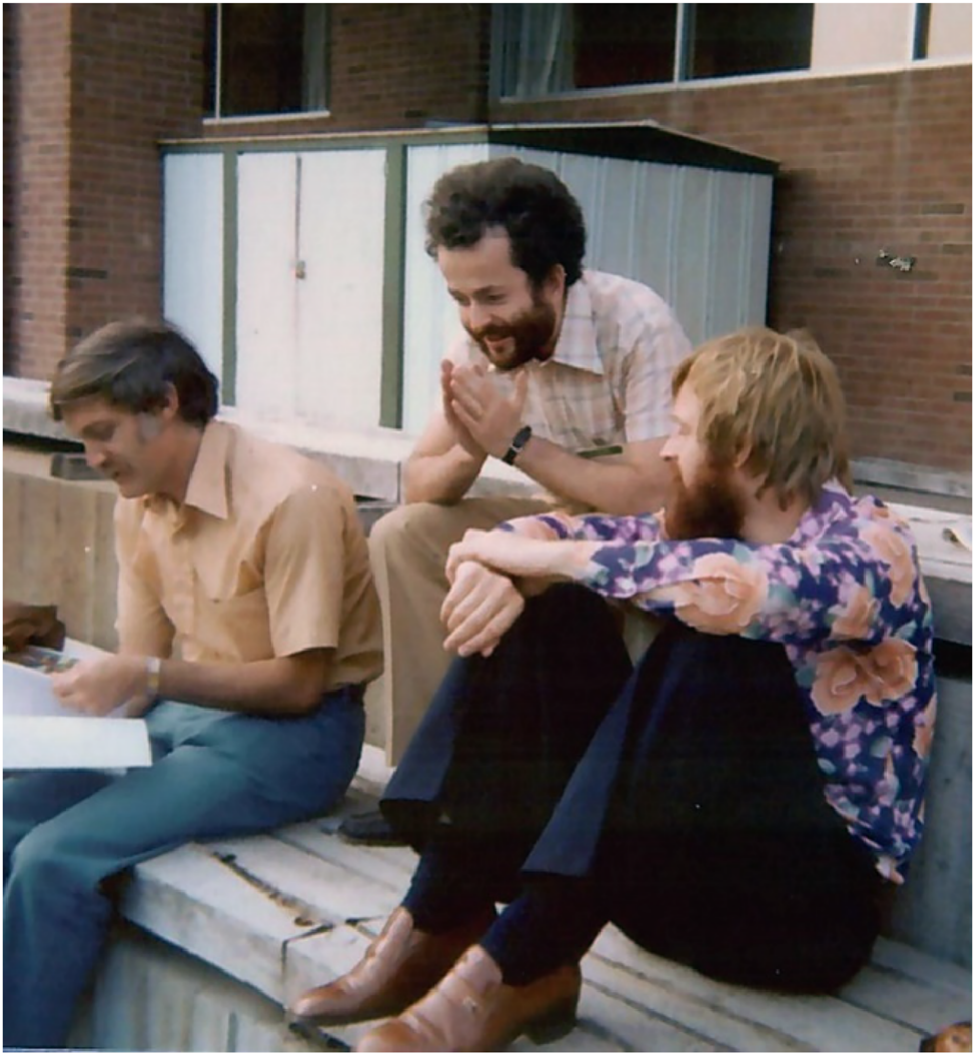


*At the 1977 DRC. From left to right:*

*Marty Pollock, Federico Capasso, Tom Pearsall*

One of my greatest joys in coming to Harvard in 2009 was rediscovering the connections with Federico and Paola, after so long a time. Paola appears to never forget the people who have touched her life and Federico’s: this is so important for setting achievements in the appropriate context. Federico’s prolific creativity and achievements are well known: while making fundamental contributions to a variety of fields in science and engineering, he has laid the foundations for the huge area of “metasurfaces” that has inspired so many other researchers. What I have truly enjoyed learning about is Federico’s mentorship and training of the next generation of researchers, of all ages. I feel so fortunate to be able to serve on the doctoral committees of many of his students, to learn about their work, but also to understand Federico’s contributions through a different lens. I see his insistence on foundational understanding, on choosing important problems now being conveyed to his students and to their students in turn. Federico’s contributions extend far beyond the publications that he has co-authored: they comprise the large communities that he has inspired and mentored.

**Mikhail Kats**

*University of Wisconsin–Madison, USA*

On this occasion to celebrate Federico’s career ‘til now, I went back to read the acknowledgements in my PhD thesis – to see what I wrote in 2013 when graduating from his group. There, I wrote about Federico’s “unbridled, nearly limitless enthusiasm for all areas of science and technology, a drive to work harder than even his first-year graduate students and postdocs, and a lack of fear of being wrong”. I have tried to develop these traits in myself, and I have been fortunate to continue to have outstanding colleagues and collaborators throughout my career.

But I do still miss working with Federico, and the environment he built in his research group. In particular, I miss the boisterous arguments! The scientific debates that would start in some meeting, and crescendo over late-night emails and spill over into the next day – always in good faith and in the spirit of discovery and scientific understanding, even if heated in the moment. I am reminded of one particularly loud debate, about the interpretation of momentum of light falling on “interfaces with a phase gradient”, today known as optical metasurfaces – that eventually led to competing word documents and pdfs furiously exchanged over emails at all hours of the night. I was too young to have had the chance to experience the environment of Bell Labs, but through Federico I felt like I could touch a piece of it.

In honor of the team Federico assembled and the environment he cultivated, I am submitting a paper we wrote in 2024, co-authored with Nanfang Yu, another of Federico’s former students and now a professor at Columbia. Nanfang was one of my first mentors in Federico’s group and a wonderful collaborator, but we had not worked together for about a decade. In Federico’s honor, we collaborate again!

**Figure:**
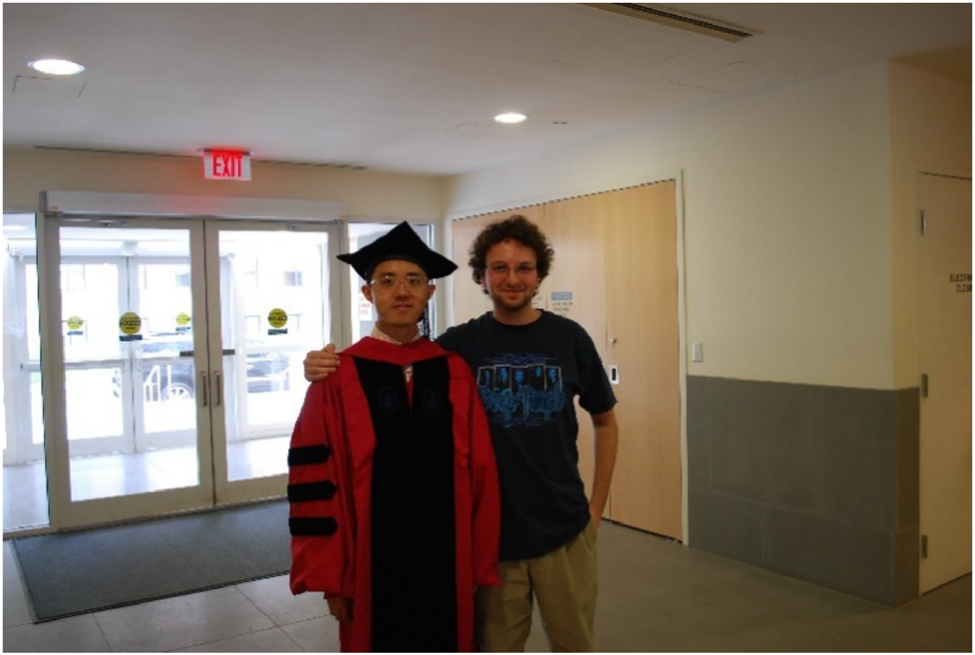


*Mikhail Kats and Nanfang Yu, two of Federico’s former students, at Nanfang’s commencement in 2009.*

**Figure:**
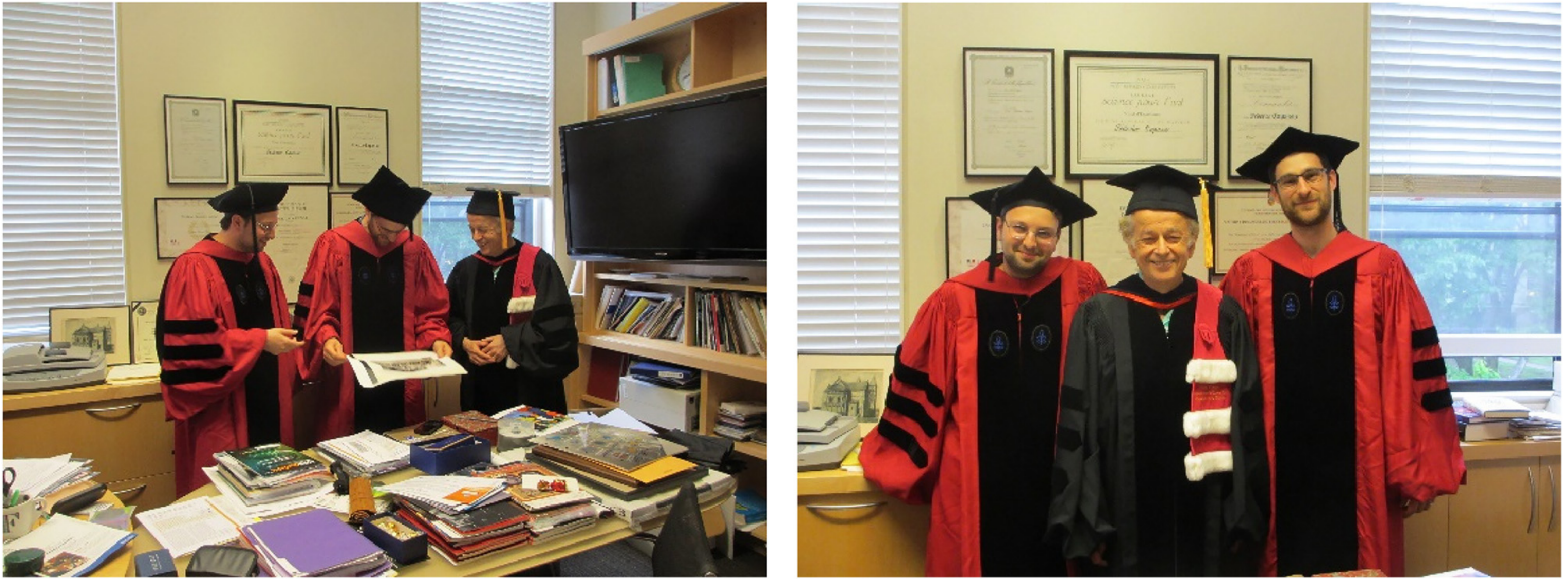


*Federico, Mikhail Kats, and Romain Blanchard in Federico’s office, after Mikhail and Romain’s commencement in 2014.*

**Figure:**
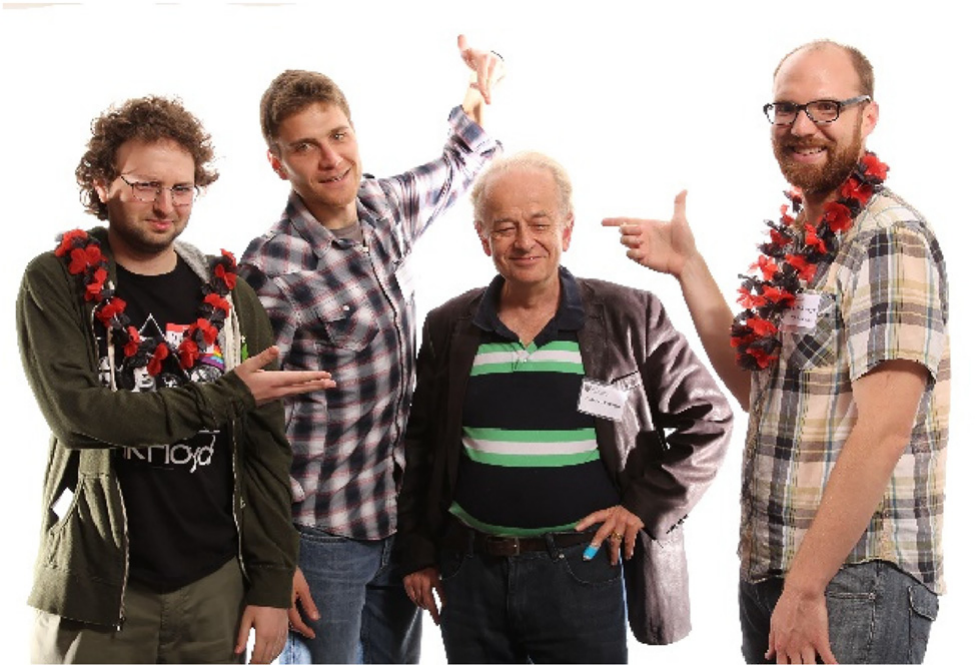


*Left to right: Mikhail Kats, Francesco Aieta, Federico, and David Woolf. All three are former PhD students.*

**Figure:**
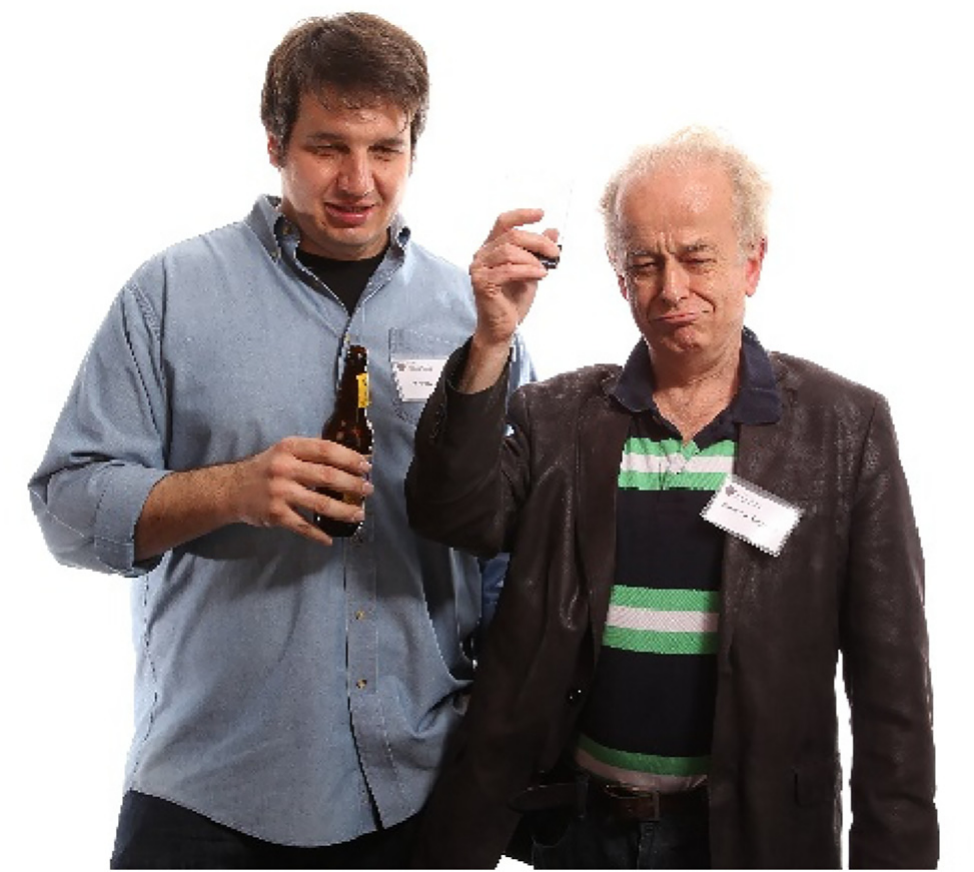


*Federico and Marko Loncar, former postdoc and now professor at Harvard.*

**Jacob Khurgin**

*Johns Hopkins University, USA*

*The year was 1984 – but this story is not Orwellian.*

At the time, I was a young mechanical engineer at Philips Research Labs, quite content working on ways to improve TV sets, lightbulbs, and small appliances like shavers and coffeemakers. One day, my boss called me into his office and said he was launching a new project on developing laser TVs powered by semiconductor lasers, and he wanted me to be part of it.

“But what is a semiconductor?” I asked.

“Interesting question,” he replied. “Why don’t you go to the International Conference on the Physics of Semiconductors in San Francisco and find out?”

So off I went.

As soon as I got hold of the conference program, I realized I was in deep water – not only did I not understand the abstracts, I didn’t even recognize half the words in the titles. I felt totally intimidated. Clearly, I thought, as a lowly engineer, I was not cut out to master physics.

Then something caught my eye: a talk with the intriguing title *Bandgap Engineering* by Federico Capasso et al. “Well,” I thought, “that says *engineering*, not science. Maybe there’s a place for me in this after all – maybe I could become a ‘bandgap engineer’?”

So I went to the talk. And there he was – a very enthusiastic, bearded speaker who didn’t seem intimidating in the least. I was completely fascinated. That evening, I barely slept.

That talk was a transformative experience. To make a long story short, a few years later I had completed my PhD and left the world of small appliances behind for a life in academia and research in semiconductors and photonics.

I’m fairly certain the world of coffee machines and shavers didn’t lose much by my departure – and I’m not sure how much the world of condensed matter and photonics gained – but what I *do* know is that Federico’s talk set me on a path that led to nearly 40 years of exciting research and interactions with brilliant, fascinating people.

For that, thank you, Federico. And happy 75th!

**Bernard Kress**

*Director, XR engineering, Google, Mountain View, CA*

**Figure:**
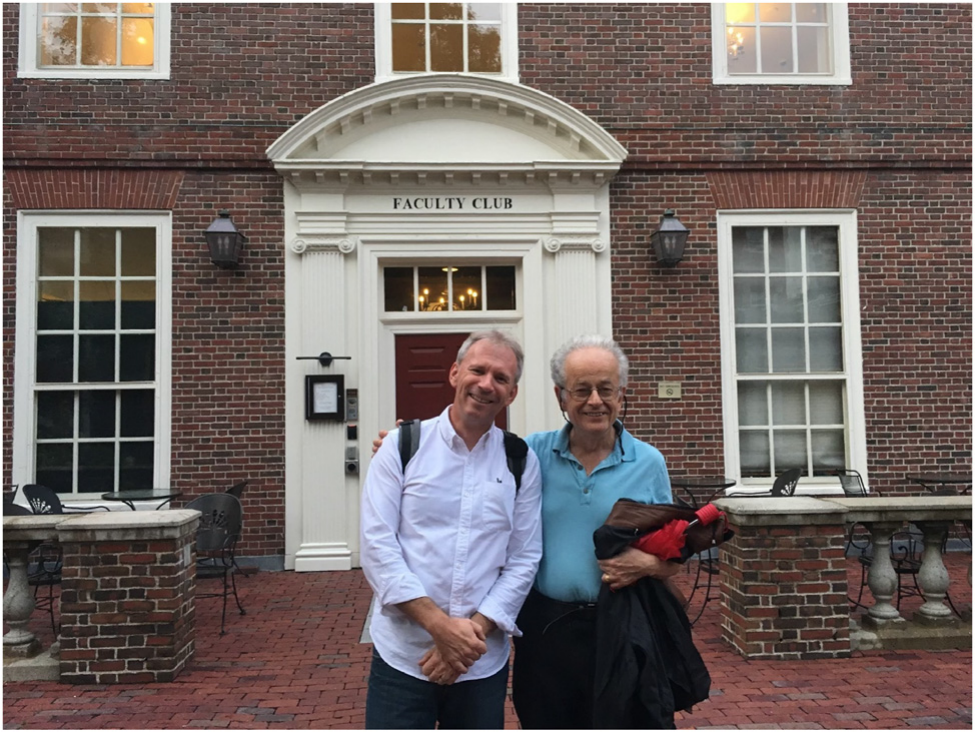


**Franco Nori**

*University of Michigan, USA*

As a great scientist and a wonderful person, Federico Capasso has been an inspiration for me over the past several decades.

One example, among many other ones follows. This experiment [5] (on Three-Dimensional Measurement of the Helicity-Dependent Forces on a Mie Particle) is a tour de force, which was based on our earlier theoretical predictions. The matching between theory and experiments was excellent, and both groups (Federico’s and mine) were very pleased.

We had shown that a Mie particle in an evanescent field ought to experience optical forces that depend on the helicity of the totally internally reflected beam. However, a direct measurement of such helicity-dependent forces had been elusive, as the widely differing force magnitudes in the three spatial dimensions place stringent demands on a measurement’s sensitivity and range. In Ref. [5], Federico’s group reported the simultaneous measurement of all components of this polarization-dependent optical force by using a 3D force spectroscopy technique with femtonewton sensitivity. The vector force fields were compared quantitatively with theoretical calculations as the polarization state of the incident light was varied and they showed excellent agreement. By plotting the 3D motion of the Mie particle in response to the switched force field, they offered visual evidence of the effect of spin momentum on the Poynting vector of an evanescent optical field.

We collaborated in another (very different) project. We analyzed [6] the use of layered superconductors as strongly anisotropic metamaterials, which can possess negative-refractive-index in a wide frequency range. Superconductors are of particular interest because they have the potential to support low losses, which is critical for applications such as super-resolution imaging. The initial idea was very appealing. However, our results indicated that it is difficult to enhance the evanescent modes in either low-*Tc* or high-*Tc* superconductors.

Every interaction with Federico was memorable and insightful. I wish we were located closer to each other and interact more closely. I wish him the very best, for a long and healthy life. We all benefit from his wisdom and insights.

**Lucia Petti**

*Institute of Applied Sciences and Intelligent Systems of CNR (ISASI-CNR), Italy*

**Figure:**
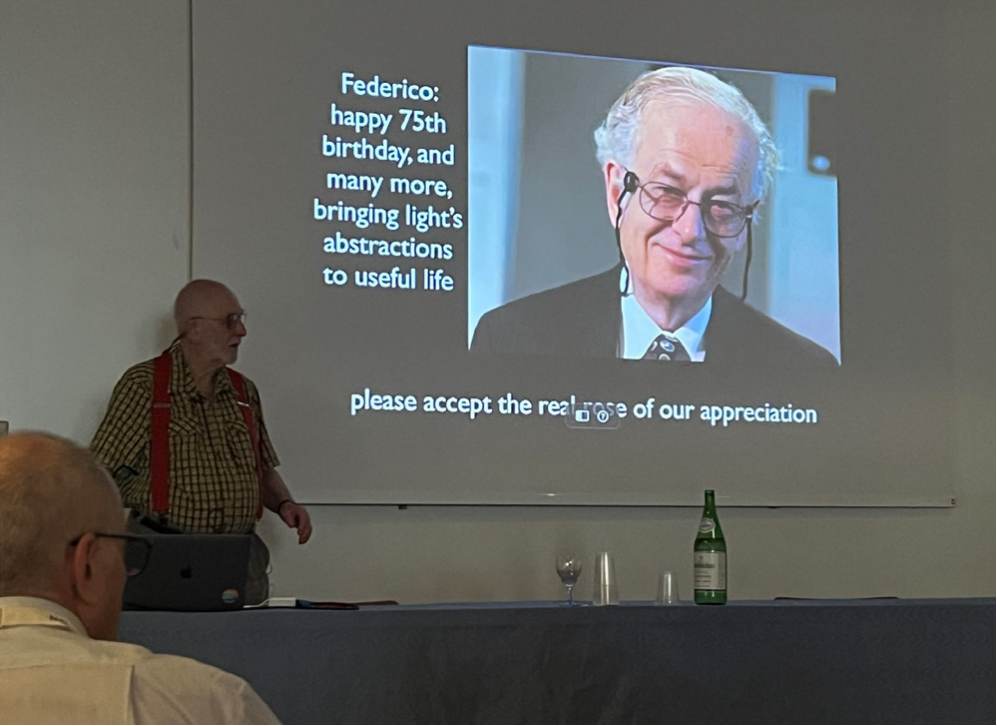


**Figure:**
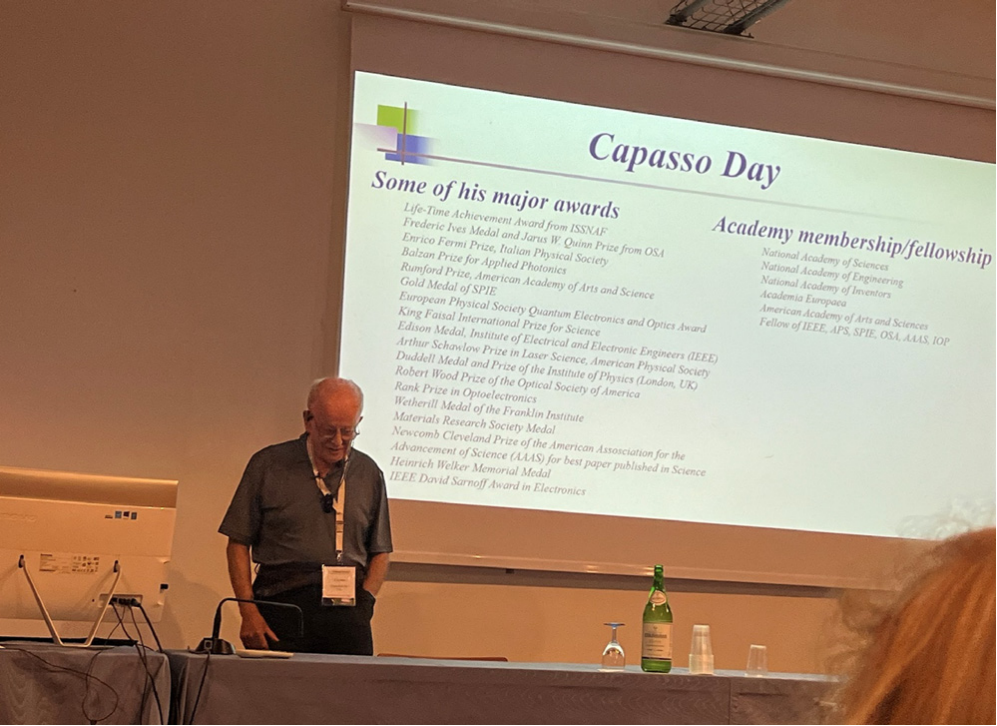


**Figure:**
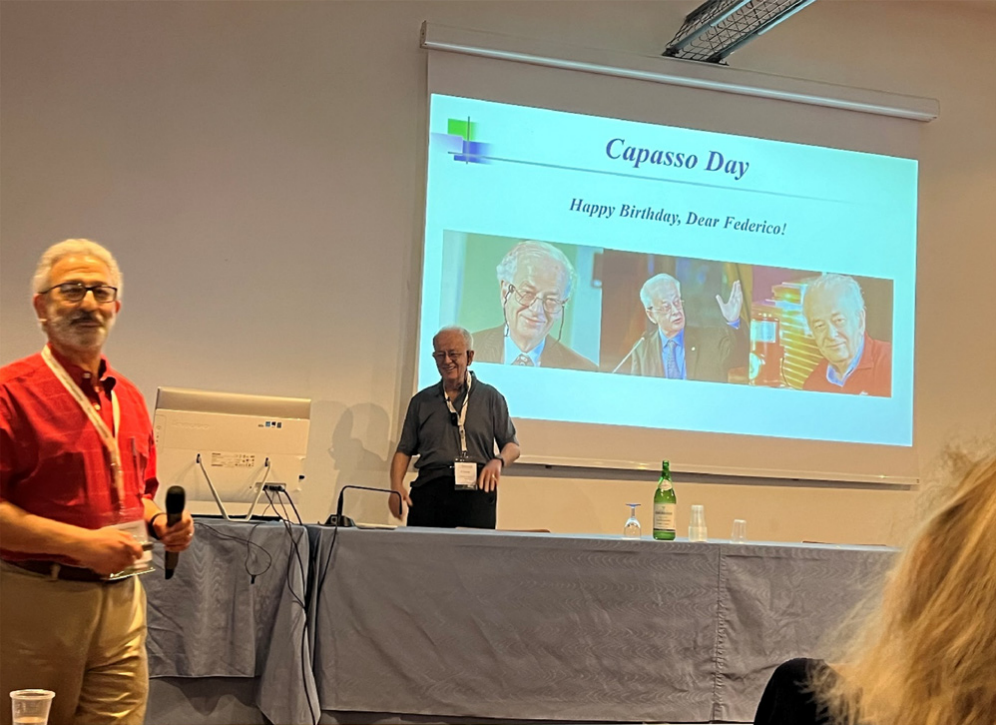


**Figure:**
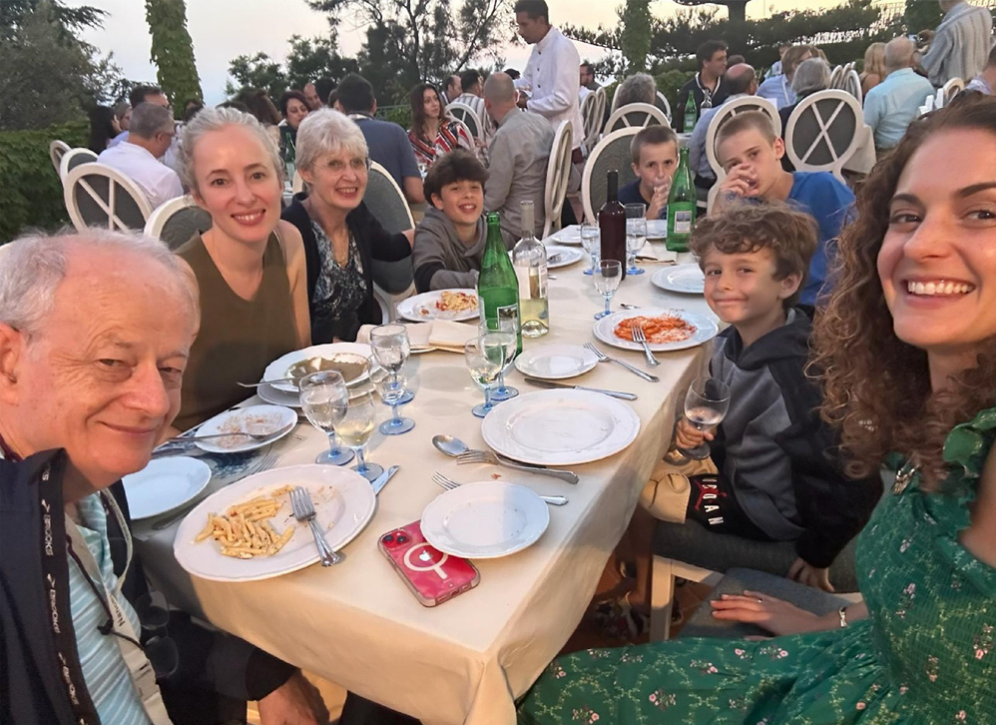


**Moti Segev**

*Technion, Israel*

**Figure:**
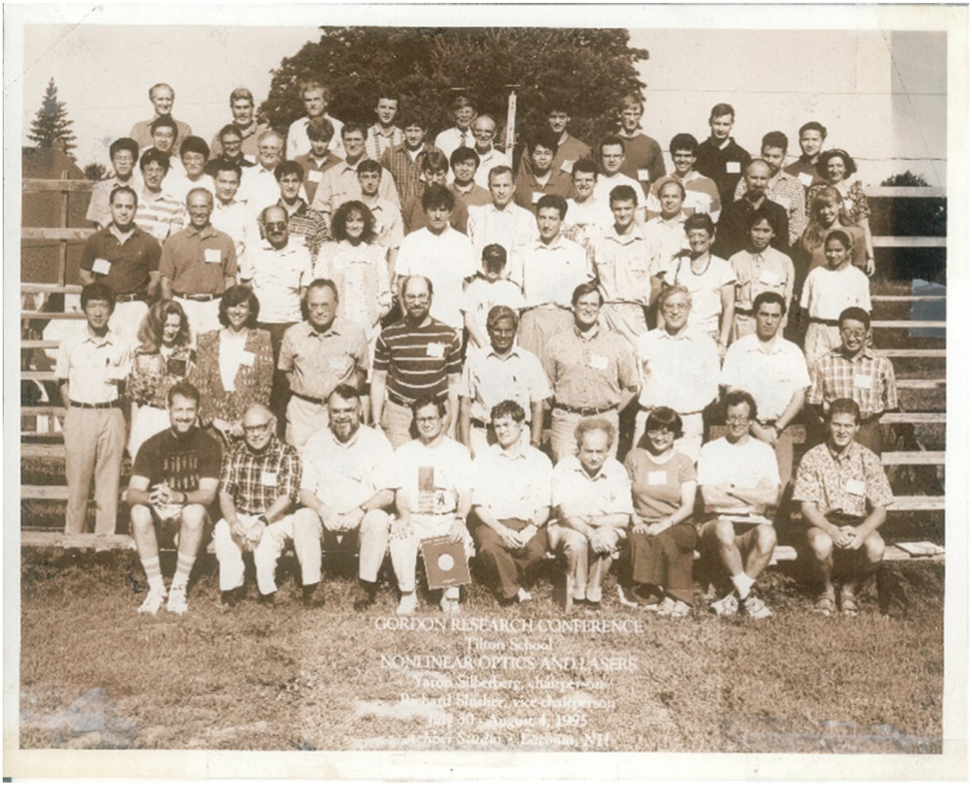


**Marlan Scully**

*Texas A&M University, USA*

**Figure:**
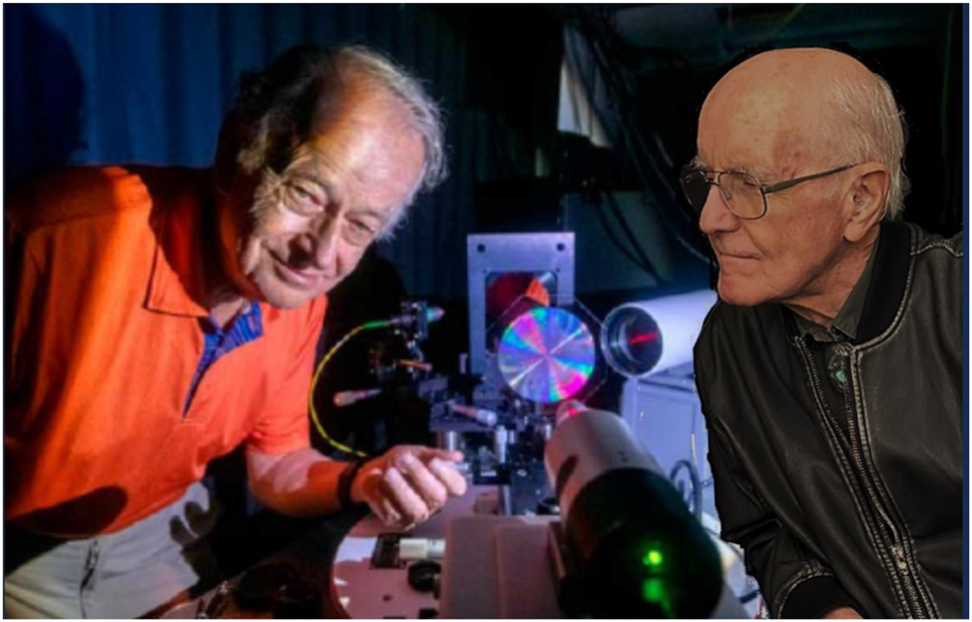


**Eli Yablonovitch**

*University of California Berkeley*

When did I first meet Federico Capasso? 1982!

**Figure:**
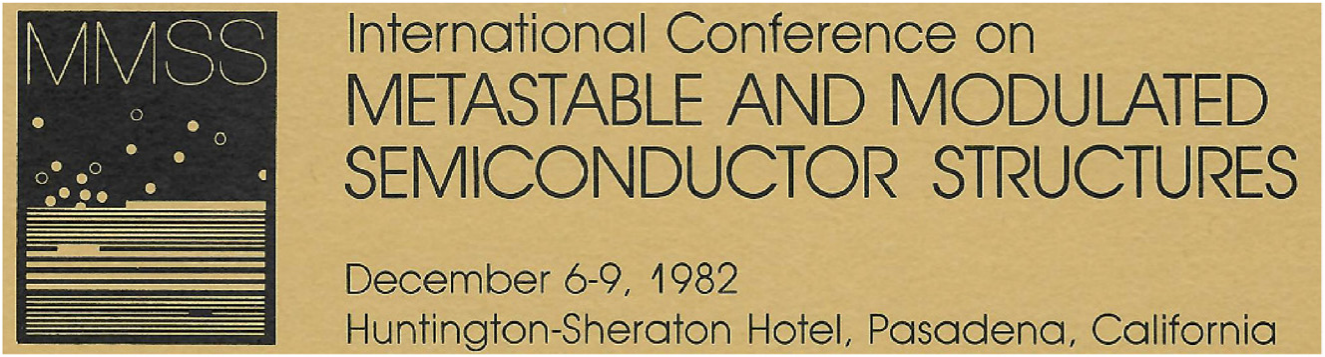


Introduced many fundamental III-V material breakthroughs, normalized MOCVD, normalized lattice mis-match, and normalized semiconductor strain.

Organized by Anupam Madhukar, USC and Frank Grunthaner, JPL.
